# Total Protein–Chloride Ratio in Pleural Fluid Independently Predicts Overall Survival in Malignant Pleural Effusion at the First Diagnosis

**DOI:** 10.3389/fonc.2021.777930

**Published:** 2022-01-10

**Authors:** Xin Qiao, Zhi-Rong Zhang, Xin-Yu Shi, Feng-Shuang Yi

**Affiliations:** ^1^ Department of Respiratory and Critical Care Medicine, Beijing Institute of Respiratory Medicine and Beijing Chao-Yang Hospital, Clinical Center for Pleural Diseases, Capital Medical University, Beijing, China; ^2^ Department of Thoracic Surgery, Beijing Chao-Yang Hospital, Capital Medical University, Beijing, China

**Keywords:** malignant pleural effusion, total protein (TP), chloride (Cl^-^), overall survival, prognostic factor

## Abstract

**Objective:**

Pre-treatment biomarkers to estimate overall survival (OS) for malignant pleural effusion (MPE) are unidentified, especially those in pleural fluid. We evaluated the relationship between OS and total protein–chloride ratio in malignant pleural effusion (PE TPClR).

**Materials and Methods:**

A retrospective study was undertaken to identify patients from 2006 to 2018 who had pathologically or cytologically confirmed MPE and received no tumor-targeted therapy. We recorded the pre-treatment clinicopathologic characteristics and follow-up status. OS was estimated by the Kaplan–Meier method, and the association between variables and OS was evaluated by Cox proportional hazards models.

**Results:**

We screened 214 patients who met the eligibility criteria. The optimal cutoff value for the PE TPClR was set at 0.53. The univariate analysis showed that there was a significant correlation between PE TPClR and OS (*P* < 0.001). The multivariate analysis between OS and the variables selected from the univariate analysis showed that the levels of neutrophil, alkaline phosphatase, neuron-specific enolase, platelets, albumin in peripheral blood, and white blood cells in pleural effusion were also independent predictors of OS.

**Conclusion:**

In patients with MPE, pre-treatment PE TPClR independently predicts OS. Although further research is necessary to generalize our results, this information will help clinicians and patients to determine the most appropriate treatment for MPE patients.

## Introduction

Malignant pleural effusion (MPE) is the second leading cause (only after parapneumonic effusion) of exudative pleural effusion, with more than 125,000 patients hospitalized annually in the United States and with an estimated hospitalization cost of over $5 billion each year (1). At present, the global incidence of MPE is affected by the elevated incidence of malignant tumors and the improvement of systematic treatment, which leads to high medical expenses (2). Despite improvements in cancer treatment, the therapy of MPE remains palliative (3), and the presence of MPE still implies a reduced survival rate (4). The median survival after the diagnosis of MPE will be affected by the origin, histological type, and stage of the primary tumor and is usually 3 to 12 months. Compared with patients who receive current standard care, advanced cancer patients receiving early palliative treatment had more improvements in the quality of life, longer survival, and less aggressive care at the end of life (5). Obviously, accurate prognosis can help to identify patients with the worst prognosis, determine appropriate treatments, and minimize unnecessary treatments and discomfort in the final stages of life.

Systemic and local inflammatory states have been considered to be associated with outcomes of cancer, and the pattern of inflammatory cell infiltration seems to affect survival (6, 7). Recently, some studies have reported various biomarkers in peripheral blood (4, 8–10) or some clinicopathological factors (11, 12) that may predict the prognosis of MPE patients or have analyzed the correlation of intrapleural immunomodulatory responses (8–10, 13–18), but simple parameters in pleural effusion have been rarely tested as possible predictors for survival in MPE patients.

It is well known that exudate is associated with inflammation or malignant processes resulting in increased capillary permeability. It is formed by active secretions or leakage and contains a high level of protein. As a result, the level of protein in pleural effusion has been studied as a simple marker for local inflammatory response (10, 14). Chloride is a biochemical marker commonly used for pleural fluid. It can be used to distinguish the etiology of pleural effusion which will significantly increase to support the diagnosis of infection, but it has not been paid enough attention in scientific research.

In our study, we comprehensively evaluated the prognostic value of clinical and laboratory characteristics in MPE patients before the tumor targeted therapy was taken, especially the markers in pleural effusion, determined the median survival time, and evaluated the prognostic variables associated with OS in MPE patients.

## Materials and Methods

### Participants

A retrospective analysis of a prospectively maintained database was carried out on identifying all patients who were diagnosed according to cancer cells in pleural effusion or pleural biopsy between June 2006 and April 2018 at Beijing Chao-Yang Hospital. In order to avoid the influence of immune-related issues and treatments on various indicators, patients with significant infection, autoimmune disease, or underlying hematological diseases, without pre-treatment complete blood or pleural count values, and who had received anticoagulant or tumor-targeted therapy were excluded. A total of 214 patients met the criteria for admission and were incorporated into our study. The research protocol was approved by the Institutional Review Board of Beijing Chao-Yang Hospital.

### Data Collection and Follow-Up

The demographic data of the patients and their clinicopathological characteristics, including laboratory variables of peripheral blood and pleural effusion, histologic type, and extrapleural metastasis, were retrospectively collected from the database collection of our institution. The patients were routinely followed up every 6 months during the first year after diagnosis. Thereafter, the follow-up should be performed annually and kept for at least 1 year. The follow-up was maintained through personal contact with patients by phone, including asking for information about tumor recurrence and survival status. OS, defined as the interval between the diagnostic date and the date of death or last follow-up, was recorded.

### Statistical Analyses

The PE TPClR values were obtained by dividing the level of total protein by the level of chloride in pleural effusion obtained during diagnostic thoracentesis or thoracoscopy. The optimal cutoff value for age was determined by the median, and those of the other variables were determined by maximally selected rank statistics (8), which were analyzed using R software, version 3.5.1, and the “maxstat” package. The cutoff value for the PE TPClR was 0.53. Continuous data were expressed as mean ± standard deviation. Categorical data were expressed by the frequency and percentage. Continuous data were analyzed by using Student’s *t*-test or one-way analysis, and categorical data were analyzed by using *χ* ^2^ test. Survival curves were analyzed by the Kaplan–Meier method, and differences were compared using the log-rank test. The relationship between the PE TPClR and survival was assessed by univariable and multivariable Cox regression models. All statistically significant univariates were included in the multivariable model. Univariate and multivariate Cox regression models provided HR and 95% CI, respectively. A Cox proportional hazards model was used to fit all individual prognostic variables to determine their independent factors. SPSS 23.0 software was used for statistical analysis. The sample power of the study was calculated by PASS 11.0.

## Results

### Patient Characteristics

The study cohort was comprised of 214 patients, and the sample power of our study reached up to 86.3%, in accordance with the two-sided log-rank test. The significance level was 0.05. The baseline characteristics of the population in this study are shown in **Table 1**. The median age of the patients was 65 years (range, 56–73 years), and there are 109 (50.9%) male and 105 (49.1%) female. The majority of patients had an ECOG PS of 0–2 (56.1%), were never smokers (61.2%), and exhibited pulmonary adenocarcinoma histology (57.5%). At the time of diagnosis, 55.1% of the patients had extrapleural metastases.

**Table 1:** Baseline characteristics of the study population according to PE TPClR.

Characteristic	All patients (*n* = 214)	PE TPClR		*P*-value
		≤0.53 (*n* = 188)	>0.53 (*n* = 26)	
OS (months), median (IQR)	15 (12, 17)	16 (13, 19)	8 (5, 11)	<0.001
Age (years), median (IQR)	65 (56, 73)	65 (56, 73)	64 (53, 75)	0.778
Sex, *N* (%)				0.348
Male	109 (50.9)	98 (52.1)	11 (42.3)
Female	105 (49.1)	90 (47.9)	15 (57.7)
ECOG PS, *N* (%)				0.549
0–2	120 (56.1)	104 (55.3)	16 (61.5)
3–4	94 (43.9)	84 (44.7)	10 (38.5)
Smoking status, *N* (%)				0.371
Ever/current	83 (38.8)	75 (39.9)	8 (30.8)
Never	131 (61.2)	113 (60.1)	18 (59.2)
Histology, *N* (%)				0.698
ADC	123 (57.5)	106 (56.4)	17 (65.4)
SQC	10 (4.7)	9 (4.8)	1 (3.8)
SCLC	10 (4.7)	9 (4.8)	1 (3.8)
Mesothelioma	13 (6.1)	13 (6.9)	0 (0)
Others	58 (27.0)	51 (27.1)	7 (27.0)
Extrapleural metastasis, *N* (%)				0.887
Yes	118 (55.1)	104 (55.3)	14 (53.8)
No	96 (44.9)	84 (44.7)	12 (46.2)
WBC, ×10^9^/L, median (IQR)	6.93 (5.76, 8.33)	6.92 (5.72,8.35)	7.25 (6.16, 7.98)	0.604
N, ×10^9^/L, median (IQR)	4.69 (3.56, 5.83)	4.60 (3.59, 5.80)	5.10 (3.40, 6.00)	0.685
Hb (g/L, M ± SD)	129.53 ± 17.30	129.64 ± 17.46	128.69 ± 16.37	0.793
ALB [g/L, median (IQR)]	34.10 (31.20, 37.13)	33.90 (30.90, 36.58)	36.65 (33.40, 38.28)	0.003
LDH [U/L, median (IQR)]	195.00 (164.75, 240.25)	199.00 (165.00, 243.00)	179.50 (161.00, 206.00)	0.179
Ca^2+^, [mmol/L, median (IQR)]	2.14 (2.07, 2.23)	2.13 (2.06, 2.22)	2.22 (2.15, 2.29)	0.001
PE ADA [U/L, median (IQR)]	13.00 (9.00, 17.00)	13.00 (9.00, 16.00)	16.00 (11.75, 26.00)	0.002
PE LDH [U/L, median (IQR)]	354.50 (197.50, 577.25)	339.50 (186.25, 521.50)	469.50 (263.00, 1,111.75)	0.024
NLR, median (IQR)	3.25 (2.11, 4.53)	3.25 (2.18, 4.52)	3.42 (1.78, 4.90)	0.824
PLR, median (IQR)	183.17 (131.70, 244.82)	183.39 (134.08, 244.52)	172.42 (116.98, 246.85)	0.495
LMR, median (IQR)	3.04 (2.17, 4.49)	3.06 (2.19, 4.38)	2.99 (2.10, 5.67)	0.495
CAR, median (IQR)	0.04 (0.15, 0.96)	0.04 (0.16, 0.95)	0.04 (0.01, 0.15)	0.584
PE TPClR, median (IQR)	0.44 (0.40, 0.48)	0.43 (0.39, 0.47)	0.59 (0.55, 0.62)	<0.001

OS, overall survival; ECOG PS, Eastern Cooperative Oncology Group performance status; ADC, adenocarcinoma; SQC, squamous cell carcinoma; SCLC, small cell carcinoma; WBC, white blood cell; N, neutrophil; Hb, hemoglobin; M ± SD, mean ± standard deviation; ALB, albumin; LDH, lactic dehydrogenase; Ca^2+^, calcium ion; PE, pleural effusion; ADA, adenosine deaminase; NLR, neutrophil to lymphocyte ratio; LMR, lymphocyte to monocyte ratio; CAR, C-reactive protein to albumin ratio; TPClR, total protein–chloride ratio.

### Association of the Pretreatment PE TPClR With Baseline Clinical Factors in MPE

According to the best cutoff value of PE TPClR, all patients were classified into low group or high group. The clinical and laboratory characteristics related to the two groups are shown in **Table 1**. The median and interquartile range of the PE TPClR were 0.43 (0.39, 0.47) and 0.59 (0.55, 0.62), respectively. Age, sex, ECOG PS, smoking status, histology, extrapleural metastasis, white blood cell (WBC), neutrophil (N), hemoglobin, lactic dehydrogenase (LDH), neutrophil to lymphocyte ratio (NLR), platelet to lymphocyte ratio, lymphocyte to monocyte ratio (LMR), and C-reactive protein to albumin ratio (CAR) had no significant difference between the two groups. However, besides the PE TPClR (*P* < 0.001), the levels of albumin (ALB; *P* = 0.003), calcium (*P* = 0.001), PE adenosine deaminase (*P* = 0.002), and PE LDH (*P* = 0.024) exhibited significant differences between the two groups.

### Association of Clinicopathological Factors and OS

The univariate associations between clinicopathologic factors and OS are shown in **Table 2**. Small cell carcinoma-type histology, high levels of WBC, N, alkaline phosphatase (ALP), and C-reactive protein (CRP), erythrocyte sedimentation rate, carcino-embryonic antigen, squamous cell carcinoma antigen, neuron-specific enolase (NSE) in peripheral blood, LDH, total protein (TP), glucose in pleural effusion, NLR, CAR, PE TPClR, low level of lymphocytes, mean platelet volume, and ALB in peripheral blood, CL^-^ in pleural effusion, and LMR were significantly related to worse outcomes (all *P <*0.05).

**Table 2:** Univariate analysis of potential associations between patient characteristics and OS.

Variable	HR	95%CI	*P*-value
Age, years			
≤65	1.00		
>65	1.027	0.748, 1.410	0.868
Sex			
Female	1.00		
Male	1.270	0.924, 1.745	0.140
ECOG PS			
0–2	1.00		
3–4	1.349	0.979, 1.860	0.068
Smoking habit			
Never	1.00		
Ever/current	1.272	0.924, 1.752	0.141
Histology			
ADC *vs*.	1.042	0.754, 1.441	0.803
SQC *vs*.	1.170	0.547, 2.501	0.686
SCLC *vs*.	2.270	1.153, 4.470	0.018
Mesothelioma *vs*.	0.470	0.220, 1.004	0.051
Extrapleural metastasis			
No	1.00		
Yes	1.303	0.950, 1.789	0.101
WBC, ×10^9^/L			
≤5.03	1.00		
s>5.03	2.242	1.292, 3.891	0.004
N, ×10^9^/L			
≤3.69	1.00		
>3.69	2.115	1.446, 3.093	<0.001
L, ×10^9^/L			
≤1.26	1.00		
>1.26	0.703	0.507, 0.976	0.036
Hb, g/L			
≤116	1.00		
>116	0.754	0.525, 1.082	0.125
PLT, ×10^9^/L			
≤373	1.00		
>373	0.604	0.342, 1.066	0.082
MPV, fl			
≤9.7	1.00		
>9.7	0.721	0.521, 0.999	0.049
ALB, g/L			
≤29.6	1.00		
>29.6	0.673	0.458, 0.990	0.045
LDH, U/L			
≤176	1.00		
>176	1.261	0.900, 1.768	0.178
ALP, U/L			
≤65	1.00		
>65	1.871	1.156, 3.028	0.011
Ca^2+^, mmol/L			
≤2.00	1.00		
>2.00	0.686	0.440, 1.070	0.097
FIB, mg/dl			
≤321.8	1.00		
>321.8	1.504	0.998, 2.226	0.051
CRP, mg/dl			
≤0.86	1.00		
>0.86	1.818	1.281, 2.579	0.001
ESR, mm/h			
≤12	1.00		
>12	1.651	1.157, 2.357	0.006
CEA, ng/ml			
≤3.49	1.00		
>3.49	1.602	1.152, 2.229	0.005
SCC, ng/ml			
≤1	1.00		
>1	1.404	1.005, 1.961	0.047
NSE, ng/ml			
≤28.9	1.00		
>28.9	2.484	1.555, 3.967	<0.001
CYFRA, ng/ml			
≤2.09	1.00		
>2.09	1.600	0.966, 2.651	0.068
PE TCs/µl			
≤2,420	1.00		
>2,420	0.739	0.469, 1.591	0.191
PE WBCs/µl			
≤383	1.00		
>383	0.647	0.411, 1.018	0.060
PE LDH, U/L			
≤155	1.00		
>155	1.960	1.182, 3.250	0.009
PE ADA, U/L			
≤19	1.00		
>19	0.776	0.502, 1.201	0.255
PE TP, g/L			
≤54.9	1.00		
>54.9	1.891	1.216, 2.941	0.005
PE Cl^-^, mmol/L			
≤109.6	1.00		
>109.6	0.419	0.249, 0.704	0.001
PE Glu, mmol/L			
≤9.28	1.00		
>9.28	1.651	1.020, 2.674	0.041
NLR			
≤1.79	1.00		
>1.79	1.941	1.210, 3.114	0.006
LMR			
≤5.71	1.00		
>5.71	0.489	0.270, 0.883	0.018
PLR			
≤181.63	1.00		
>181.63	1.301	0.947, 1.790	0.105
CAR			
≤0.03	1.00		
>0.03	1.786	1.265, 2.521	0.001
PE TPClR			
≤0.53	1.00		
>0.53	2.302	1.498, 3.535	<0.001

OS, overall survival; ECOG PS, Eastern Cooperative Oncology Group performance status; ADC, adenocarcinoma; SQC, squamous cell carcinoma; SCLC, small cell carcinoma; WBC, white blood cell; N, neutrophil; L, lymphocyte; Hb, hemoglobin; PLT, platelet; MPV, mean palate volume; ALB, albumin; LDH, lactic dehydrogenase; ALP, alkaline phosphatase; Ca^2+^, calcium ion; FIB, fibrinogen; CRP, C-reactive protein; ESR, erythrocyte sedimentation rate; CEA, carcino-embryonic antigen; SCC, squamous cell carcinoma antigen; NSE, neuron-specific enolase; CYFRA, cytokeratin 19 fragment; PE, pleural effusion; TCs, total cells; ADA, adenosine deaminase; TP, total protein; Cl, chloride; Glu, glucose; NLR, neutrophil to lymphocyte ratio; LMR, lymphocyte to monocyte ratio; PLR, platelet to lymphocyte ratio; CAR, C-reactive protein to albumin ratio; TPClR, total protein–chloride ratio.

### PE TPClR Predicted OS Independently

**Figure 1:**
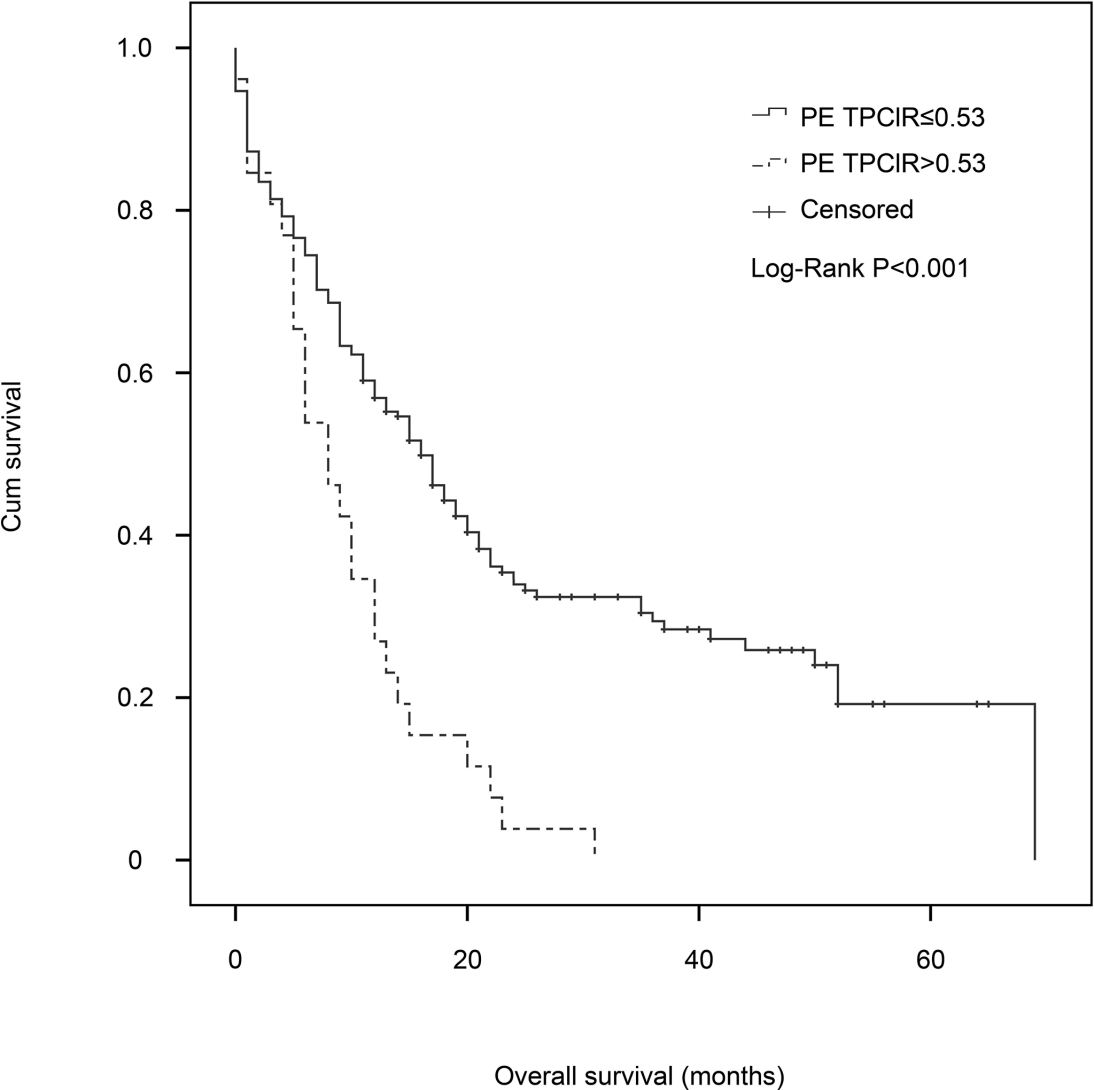
Kaplan–Meier estimates of overall survival for patients with malignant pleural effusion in the pleural effusion total protein–chloride ratio (PE TPClR) ≤0.53 and PE TPClR >0.53 groups. There was a significant difference in survival between the groups (*P* < 0.001; stratified log-rank test).

Finally, we conducted a multivariate Cox analysis to determine whether PE TPClR independently predicted OS or not (**Table 3**). After adjusting, we found that pre-treatment PE TPClR was associated with OS independently (HR, 3.182; 95% CI, 2.203–5.003; *P* < 0.001). The median survival time was significantly higher for patients with PE TPClR ≤0.53 than for those with TPClR >0.53 (16 *vs*. 8 months; *P <*0.001), as shown in **Figure 1**. N (*P* < 0.001), platelet (*P* = 0.004), ALB (*P* = 0.024), ALP (*P* = 0.005), NSE (*P* < 0.001), and PE WBC (*P* = 0.001) were also independently correlated with OS. Analyses including pre-treatment WBC, NLR, LMR, and CAR in the multivariate model are shown above, but they were not significantly related to OS (*P* = 0.381, *P* = 0.541, *P* = 0.471, and *P* = 0.167, respectively).

**Table 3:** Multivariate analysis of potential associations between patient characteristics and OS.

Variable	HR	95%CI	*P*-value
N, ×10^9^/L			
≤3.69	1.00		
>3.69	2.310	1.560, 3.419	<0.001
PLT, ×10^9^/L			
≤373	1.00		
>373	0.422	0.234, 0.760	0.004
ALB, g/L			
≤29.6	1.00		
>29.6	0.633	0.426, 0.941	0.024
ALP, U/L			
≤65	1.00		
>65	2.047	1.244, 3.368	0.005
NSE, ng/ml			
≤28.9	1.00		
>28.9	2.880	1.750, 4.739	<0.001
PE WBCs/µl			
≤383	1.00		
>383	0.436	0.271, 0.703	0.001
PE TPClR			
≤0.53	1.00		
>0.53	3.182	2.203, 5.003	<0.001

OS, overall survival; N, neutrophil; PLT, platelet; ALB, albumin; ALP, alkaline phosphatase; NSE, neuron-specific enolase, PE, pleural effusion; WBCs, white blood cells; TPClR, total protein–chloride ratio.

## Discussion

In our study, we have extensively screened out commonly used clinical laboratory indexes, which included MPE from a variety of causes that are not limited to lung cancer, and found that the PE TPClR is an important prognostic factor for OS in MPE patients. The PE TPClR is effective, safety, easy to calculate, inexpensive, and generally applicable in clinical settings from the different indicators in MPE (19). Therefore, the PE TPClR could potentially be an attractive and ideal prognostic variable for predicting the survival of MPE patients, and more valuable prognostic information can be provided for clinicians and patients through it. As far as we know, this is the first study on the effect of simultaneous detection of TP and chloride levels in pleural effusions on the prognosis of MPE.

Inflammation, which is reported to play a significant role in different stages of oncogenesis, is now considered as a marker of cancer (20). As we have mentioned above, plenty of research have explored the most commonly used indicators of inflammatory response, such as leukocytes, leukocyte subtypes, cytokines, CRP, LDH, ALP, and their potential effects on the prognosis of cancer patients. However, markers in pleural effusions are rarely used as possible predictors for survival in patients with MPE.

In our study, a higher level of total protein in pleural effusion was related to a shorter OS in the univariate analysis. It is different from some previous studies on the relationship between the total protein in pleural fluid and survival time. Abrao et al. showed that, when the pleural fluid total protein value was <3.6 g/dl, the median survival was 74 days, which was statistically significant in the multivariate analysis (14). They believe that, over time, the progress of the disease may affect hypoalbuminemia, but we think that exudative pleural effusion, which contains a high level of protein, is resulting in increased capillary permeability. When inflammation occurs, higher capillary permeability may cause the protein in exudation to increase.

There was no in-depth study of chlorides in pleural effusions except for the identification of drowning types. In our previous study (21), we found that the Cl^-^ level in pleural fluid was an independent indicator for prognosis in MPE patients. Few studies have found that chloride intracellular channels 3 and 4, controlling the intracellular distribution of Cl^-^ to provide ionic counterbalance, are over-expressed in human mesothelioma (22), and the concentration of Cl^-^ in heart-failure-associated pleural effusion is higher than that in serum, indicating that Cl^-^ may play an important role in the formation and retention of body fluid in the thoracic cavity (23). There are many studies on the level of chloride in the cerebrospinal fluid, which show that the concentration of chloride decreases when the level of chloride in the blood decreases. The pH of the cerebrospinal fluid decreases, and inflammatory exudation and adhesion are obvious. According to the existing literature, tumor cells produce hydrogen ions by glucose metabolism. Therefore, low pleural fluid pH and low glucose reflect a higher pleural tumor load and are related to poor survival. Heffner et al. confirmed that when pleural pH was less than 7.28, the prognosis of MPE was poor (24). Our results may be corroborated by the correlation between pH and chloride levels in pleural effusions. Therefore, a high level of PE TPClR was associated with a shorter OS.

The main limitation of our study is that it is a retrospective study with a relatively small sample size. Although a high statistical significance has been achieved, it is still necessary to conduct further studies on more patients. Further studies are needed to identify other sensitive biomarkers in pleural effusion to determine the best combination of marker analysis.

## Conclusion

PE TPClR can be used to predict the prognosis of MPE patients. It can help clinicians select patients for appropriate palliative care. More research is needed to clarify the underlying mechanisms and to identify new strategies to improve the prognosis of these patients.

## Data Availability Statement

The original contributions presented in the study are included in the article/supplementary material. Further inquiries can be directed to the corresponding author.

## Ethics Statement

The studies involving human participants were reviewed and approved by the ethics committee at Beijing Chao-Yang Hospital. Written informed consent for participation was not required for this study in accordance with the national legislation and institutional requirements.

## Author Contributions

XQ designed the study, did patient recruitment and assessment, collected information, analyzed the relevant data, and drafted the manuscript. Z-RZ and X-YS analyzed the relevant data and drafted the manuscript. F-SY conceived the idea, guided the study, and revised the paper critically to ensure the integrity of this research. All authors contributed to the article and approved the submitted version.

## Funding

This work was supported by grants from the National Natural Science Foundation of China (nos. 31700790 and 82000098).

## Conflict of Interest

The authors declare that the research was conducted in the absence of any commercial or financial relationships that could be construed as a potential conflict of interest.

## Publisher’s Note

All claims expressed in this article are solely those of the authors and do not necessarily represent those of their affiliated organizations, or those of the publisher, the editors and the reviewers. Any product that may be evaluated in this article, or claim that may be made by its manufacturer, is not guaranteed or endorsed by the publisher.
